# Auditory Phenotype and Histopathologic Findings of a Mutant Nlrp3 Expression Mouse Model

**DOI:** 10.3389/fneur.2022.890256

**Published:** 2022-06-24

**Authors:** Yehree Kim, Sang-Yeon Lee, Min Young Kim, Kyusun Park, Jin Hee Han, Jung Ho Kim, Bong Jik Kim, Byung Yoon Choi

**Affiliations:** ^1^Department of Otorhinolaryngology, Seoul National University Bundang Hospital, Seongnam, South Korea; ^2^Department of Otorhinolaryngology, Seoul National University Hospital, Seoul, South Korea; ^3^Department of Pathology, Seoul National University Hospital, Seoul, South Korea; ^4^Department of Otorhinolaryngology-Head and Neck Surgery, Chungnam National University, Chungnam National University Sejong Hospital, Sejong, South Korea

**Keywords:** autoinflammatory inner ear disease, Nlrp3, inflammasome, hearing loss, *Gfi1* ^*Cre*^

## Abstract

**Objective:**

The pathogenesis of hearing loss in autoinflammatory disorders due to activation of the inflammasome remains incompletely understood. Previously no animals expressing mutant Nlrp3 (NOD-, LRR- and pyrin domain-containing protein 3) survived to an age when hearing evaluation was possible due to embryonic lethality. We aimed to establish a novel mouse model that manifests quantifiable hearing loss with other syndromic features due to alteration of *Nlrp3* and investigate the audiologic and histopathologic phenotype in the cochlea to clarify how the genetic alterations of NLRP3 could induce autoinflammatory hearing loss.

**Methods:**

To induce inner ear expression of the mutant Nlrp3, *Nlrp3*^*D*301*NneoR*^ mice were bred with *Gfi1*^*Cre*^ knock-in mice for conditional mutant Nlrp3 activation in the cochlea and hematopoietic cells. Hearing thresholds were measured. Hematoxylin-eosin sections of the cochlea, brain, kidney, and liver were examined under light microscopy. Immunohistochemical analyses using polyclonal anti-NLRP3 antibodies on cochlear whole-mount preparations and frozen sections were performed.

**Results:**

We, for the first time in the literature, established a mouse model that manifests quantifiable hearing loss due to *Nlrp3* alteration. ABR recordings of *Nlrp3*^*D*301*NneoR*/+^; *Gfi1*^*Cre*/+^ mice, albeit with limited life expectancy, exhibited severe to profound hearing loss at postnatal day 20 (P20). There was overall overexpression of mutant Nlrp3, and mutant Nlrp3 expression was noted in the spiral prominence, the outer sulcus region (Claudius cells and outer sulcus cells), the organ of Corti, the inner sulcus, and the spiral ganglion neurons in the cochlea. The hematoxylin-eosin sections of *Nlrp3*^*D*301*NneoR*/+^; *Gfi1*^*Cre*/+^ mice cochleae at P12 exhibited a disorganized organ of Corti between the outer hair cells/supporting Deiters' cells and basilar membrane compared with the normal phenotype mice, leading to a collapsed Nuel's space. This morphologic feature gradually returned to normal by P15. Varying degrees of inflammation with lymphocytic infiltrations were observed in the brain, kidney, and liver.

**Conclusion:**

We report the first mutant Nlrp3 overexpression mouse model (*Nlrp3*^*D*301*NneoR*/+^; *Gfi1*^*Cre*/+^) that shows obvious overexpression of Nlrp3 in the cochlea, a transient developmental lag of the cochlea, and severe to profound hearing loss. We expect that this mouse line, which models human autoinflammatory hearing loss, could provide a valuable tool to elucidate the underlying pathogenic mechanism of inflammasome activation-mediated hearing loss.

## Introduction

The *NLRP3* (NOD-, LRR-, and pyrin domain-containing protein 3) gene encodes the NLRP3 protein, the central component of the NLRP3 inflammasome (1). The NLRP3 inflammasome is an innate immune sensor expressed in immune cells (2, 3), that leads to the secretion of interleukin-1β when activated. Activation of the NLRP3 inflammasome transforms the inactive procaspase-1 to active caspase-1 which can process pro–IL-1β to mature IL-1β (4).

Alterations in the *NLRP3* gene are associated with several autoinflammatory disorders affecting several organs, referred to as cryopyrin-associated periodic syndromes (CAPS), which include a spectrum of disorders—chronic infantile neurological cutaneous and articular syndrome (CINCA or NOMID, neonatal onset multisystem inflammatory disease), Muckle-Wells syndrome and familial cold autoinflammatory syndrome (FCAS) (5, 6).

Hearing loss is one of the presenting symptoms of these autoinflammatory disorders. About 76, 86, 33, and 25% of NOMID, NOMID/MWS, MWS, and FCAS subjects exhibit hearing loss (7). Recent reports have suggested that hearing loss could be the sole symptom in a subset of autoinflammatory diseases (DFNA34) (8, 9).

Activation of the NLRP3 inflammasome requires at least two signals—the initial priming signal and the second activation signal (10). However, in patients with pathogenic variants of NLRP3 gene, this process only requires the initial priming signal to induce IL-1β secretion(11). Anakinra, a non-glycosylated recombinant version of the endogenous human IL-1 receptor antagonist, was reported to control systemic inflammation (12). Intriguingly, it also showed partial effectiveness in the treatment of autoinflammatory hearing loss (9).

Cochlear implantation in patients with this disease entity demonstrated successful auditory rehabilitation outcomes (13). Cochlear implantation could work as a rescue measure in cases of advanced, profound *NLRP3*-related hearing loss, and an anti-IL-1β agent could be beneficial for early diagnosed, mild hearing loss. However, it has not been clearly elucidated how hearing loss develops in this disease entity; therefore, prevention or timely intervention remains a challenging issue in the management of hearing loss as a presenting symptom of autoinflammatory disorders.

A prior study identified Nlrp3 expression in normal mouse cochlear macrophage-like cells expressing Cx3cr1 (8). The authors found that the NLRP3 inflammasome could be activated with the priming and the activating signals, thus indicating that macrophage/monocyte-like cells in the cochlea can cause hearing loss through innate immune response.

To study the pathogenesis of hearing loss in autoinflammatory disorders due to activation of the inflammasome from developmental and immunological perspectives, a mouse model recapitulating human autoinflammatory disorders with quantifiable hearing loss is mandatory. However, in a previous model, severe inflammation due to *Nlrp3* mutation resulted in an extremely short life expectancy, which made an objective hearing assessment impossible.

We generated a double knock-in mouse model by breeding *Nlrp3*^*D*301*NneoR*^ knock-in mice with *Gfi1*^*Cre*^ (growth factor independent 1 transcriptional repressor) knock-in mice, a known tool for inducing inner ear hair cell-specific expression of a target gene (14–16) thereby generating the first-ever mouse model in the literature manifesting with both hearing loss and overexpression of Nlrp3. In this study, first, we conducted detailed audiologic and histopathologic phenotyping of gain of function of mutant Nlrp3 in the cochlea. By doing this, we aimed to elucidate how alterations of NLRP3 in the cochlea could play a role in the development of autoinflammatory hearing loss.

## Materials and Methods

### Animals and Housing

To generate the Nlrp3 gain-of-function model, we bred congenital-onset, multisystemic inflammatory disease mice (*Nlrp*3^D301NneoR^, obtained from The Jackson Laboratory, Jax #017971) with heterozygous *Gfi1*^*Cre*^ knock-in mice (15) to generate mice mutant (*Nlrp3* ^*D*301*NneoR*^^/+^; *Gfi*1^*Cre*^/+) and normal phenotype littermate controls (*Nlrp3* ^*D*301*NneoR*^^/+^; *Gfi*1^+^/+) on a mixed background (*Nlrp*3^D301NneoR^ was generated on a C57BL/6J background and *Gfi1*^*Cre*^ knock-in mice on 129S6 and C57BL/6J mixed background) expressing mutant Nlrp3 in the inner ear and the hematopoietic cells.

For hearing threshold measurement and histopathologic analyses, in which haploinsufficiency of *Gfi1* could affect the outcomes (15), the heterozygous *Gfi1*^*Cre*^ knock-in mice (*Nlrp*3^+^/+; *Gfi*1^*Cre*^/+) were also tested out as controls.

All animals were kept on a 12-h light-dark cycle in a specific-pathogen-free facility with controlled temperature and humidity and had free access to food and water. All experimental procedures were conducted according to protocols approved by the Institutional Animal Care and Use Committee of Seoul National University Bundang Hospital (Seongnam, Republic of Korea, IACUC No.: BA-2004-294-038-09).

### Conditional Expression of Mutant Nlrp3 in the Mouse Inner Ear

To induce inner ear expression of the mutant Nlrp3, *Nlrp3*^*D*301*NneoR*^ mice bearing a loxP-flanked neomycin-resistant cassette (reverse orientation) in intron 2 and a point mutation in exon 3 of *Nlrp3* were bred with *Gfi1*^*Cre*^ knock-in mice, generating *Nlrp3* ^*D*301*NneoR*^^/+^; *Gfi*1^*Cre*^/+ mice for conditional mutant Nlrp3 activation only in the cochlea and hematopoietic cells.

Genotyping was performed by polymerase chain reaction (PCR) on genomic DNA extracted from ear punches. The primers used for *Gfi1*^*Cre*^ genotyping were as follows: *Gfi1* wild-type forward (5′-GGG ATA ACG GAC CAG TTG-3′), *Gfi1* wild-type reverse (5′-CCG AGG GGC GTT AGG ATA-3′), and *Gfi1*^*Cre*^ reverse (5′-GCC CAA ATG TTG CTG GAT AGT-3′). The primers used for *Nlrp3* genotyping were as follows: *Nlrp3* wild-type forward (5′- CAC CCT GCA TTT TGT TG−3′), *Nlrp3* mutant forward (5′- GCT ACT TCC ATT TGT CAC GTC C−3′), and common reverse (5′- CGT GTA GCG ACT GTT GAG GT -3′).

### Auditory Brainstem Responses

The hearing threshold of 10 *Nlrp3* ^*D*301*NneoR*^^/+^; *Gfi*1^*Cre*^/+ mice (seven males and three females), six *Nlrp3* ^*D*301*NneoR*^^/+^; *Gfi*1^+^/+ mice (four males and two females), four *Nlrp*3^+/+^; *Gfi*1^*Cre*^/+ mice (two males and two females) and four *Nlrp3* ^+/+^; *Gfi*1^+/+^ mice (two males and two females) were measured at postnatal day 20 (P20). The hearing threshold of the right ear of each animal was used for analysis. For auditory brainstem response (ABR) testing, mice were anesthetized with an intraperitoneal injection of a mixture of zolazepam/tiletamine (50 mg/kg, Zoletil 50; Virbac, Carros, France) and 10 mg/kg xylazine (Rompun; Bayer Health Care, Leverkusen, Germany). Reference electrodes were attached posterior to the right pinna, a non-inverting electrode was attached at the vertex of the skull, and a ground electrode was attached to the contralateral ear (17). Hearing thresholds were determined at 4, 8, 16, and 32 kHz using a SmartEP device (Intelligent Hearing Systems, Miami, FL, USA). Acoustic stimuli were presented directly to the entrance of the ear canal. Tone burst sound stimuli were presented from 100 dB SPL and decreased 10 dB decrements until the lowest level at which a distinct ABR wave pattern could be recognized by two of the investigators. The ABR threshold was determined as this lowest recognizable ABR response. For mice with no ABR responses, the hearing threshold was calculated as 110 dB SPL. All recordings were performed in a soundproof box, and ABR was tested at postnatal day 20 (P20).

### Acquisition of Magnetic Resonance Images

To check for cochlear inflammation, pre- and post-contrast enhanced magnetic resonance imaging (MRI) was performed (*n* = 2, one *Nlrp3* ^*D*301*NneoR*^^/+^; *Gfi*1^*Cre*^/+ and one *Nlrp3* ^*D*301*NneoR*^^/+^; *Gfi*^+/+^, both male) at P15. All MR scans were performed on a MRS^*^DRYMAG 7 Tesla (T) MRI system (MR solutions, Guilford, UK) using gradients with a maximum gradient strength of 420 mT/m. Mice were anesthetized as described in the ABR section and body temperature was maintained at 37°C by circulating warm air through the magnet bore. T1-weighted images were acquired first. The mice were scanned using a two-dimensional (2D) spoiled gradient echo sequence (acquisition matrix = 256 × 256; field of view = 20 mm × 20 mm; slice thickness = 3 mm; flip angle = 90°).

Immediately after T1 measurement, the mouse was removed from the scanner, injected with a single bolus of gadolinium meglumine (Dotarem; Guerbet, Villepinte, France) at a dose of 0.2 mmol/kg through the tail vein, and then re-positioned in the scanner. Proper delivery of contrast agent was confirmed first by scanning the abdomen and checking for contrast material in the mouse bladder. Thirty minutes after the injection of contrast agent, contrast enhanced images were acquired using the same acquisition matrix as the precontrast images.

### Cochlear Extraction and Preparation of Histologic Sections

To examine the presence of any cochlear structural abnormalities, hematoxylin-eosin stain was performed at P12, P15, and P20 (*n* = 4 cochleae of *Nlrp3* ^*D*301*NneoR*^^/+^; *Gfi*1^*Cre*^/+ mice, and *n* = 4 cochleae of *Nlrp3* ^*D*301*NneoR*^^/+^; *Gfi*^+/+^ for each timepoint). Mice were anesthetized as described before in the ABR section and decapitated. Both cochleae were extracted and fixed in ice-cold 4% paraformaldehyde overnight. The fixed cochleae were washed, dehydrated, and embedded in paraffin. Serial sections were made at 5-μm thickness, attached to the slide glass, and air-dried. The tissue sections were deparaffinized, hydrated, stained with HE, dehydrated, cleared, and sealed with a cover glass. The histologic characteristics were examined under a light microscope.

### Immunohistochemistry of the Cochlear Tissue

Immunohistochemistry was performed on *Nlrp3* ^*D*301*NneoR*^^/+^; *Gfi*1^*Cre*^/+ and *Nlrp3* ^*D*301*NneoR*^^/+^; *Gfi*^+/+^ mice at P17 (*n* = 4 cochleae for each type). Cochlear turns were carefully excised, and whole-mount preparations were made and incubated in blocking/permeabilizing buffer (phosphate-buffered saline [PBS] with 5% goat serum and 0.25% Triton X-100). Cochlear frozen sections were also prepared and incubated in blocking/permeabilizing buffer. The preparations were incubated overnight at 4°C with 1:100 rabbit anti-NLRP3 (PA5-88709, Invitrogen, Waltham, MA, USA) primary polyclonal antibodies diluted in the blocking/permeabilizing buffer. After 3 washes, the cochlear turns were reacted with fluorescence-labeled 1:400 goat anti-rabbit (ab150077, Abcam, Cambridge, UK) secondary antibodies diluted in blocking/permeabilizing buffer for 2 h at room temperature. After 3 washes with PBS, the cochlear turns were reacted with 1:100 rhodamine phalloidin (R415, Invitrogen, Waltham, MA, USA) diluted in blocking/permeabilizing buffer for 1 h at room temperature. The samples were then rinsed 3 times with PBS. High-resolution images were obtained using a confocal laser scanning microscope (LSM710, Zeiss). Each image stack was reduced to a two-dimensional maximum intensity Z-projection image.

Prior to the immunohistochemistry procedures on cochlear tissues, the anti-NLRP3 antibody was validated on human embryonic kidney (HEK) 293 cells transfected with NLRP3 (Myc-DDK-tagged) cDNA (catalog no. RC220952) (Supplementary Figure 1).

### Hair Cell Count

The number of inner and outer hair cells were counted from the confocal images of the whole-mount preparations described above (Immunohistochemistry of the cochlear tissue). Serial confocal images of hair cells labeled with phalloidin were obtained from each cochlea using laser scanning confocal microscope (LSM710, Zeiss). The entire length of the organ of Corti was reconstructed by overlapping the common cells at the edges of the individual images. The reconstructed image was then divided into segments, each spanning 1% of the total length. The number of IHCs and OHCs in each segment vs. the relative distance from the apex were plotted (18).

### Histologic Examination of Other Organs

Histologic evaluation with HE staining was performed on two *Nlrp3* ^*D*301*NneoR*^^/+^; *Gfi*1^*Cre*^/+ mice and one *Nlrp3* ^*D*301*NneoR*^^/+^; *Gfi*^+/+^ mouse at P20 to check for histopathologic signs of systemic inflammation in other organs. HE-stained tissue slides of the brain, eyes, liver, spleen, stomach, kidney and legs were prepared and the findings were assessed by an experienced pathologist (JHK) with more than 15 years of diagnostic experience. The type of immune cells infiltrating each tissue was determined by the microscopic morphology.

### Data Analysis and Statistical Analysis

Data analysis was carried out using SPSS version 25.0 (IBM Corp., Armonk, NY, USA). For comparisons between two samples, the significance of data was assessed by the Student *t*-test when samples showed a normal distribution or by the Mann-Whitney test when samples did not pass the normality test. Comparisons of more than 2 samples were conducted using one-way analysis of variance with the Tukey *post-hoc* test when data passed the normality test or the Kruskal-Wallis test with the Dunn's *post-hoc* test when data did show a normal distribution. A *P*-value of < 0.05 was considered to indicate statistical significance.

## Results

### Auditory Brainstem Responses Reveal Profound Deafness in *Nlrp3 ^*D*301*NneoR*^* ^/+^; *Gfi*1^*Cre*^/+ Mice

The hearing phenotype was assessed at P20 by ABR recordings, which revealed that *Nlrp3* ^*D*301*NneoR*^^/+^; *Gfi*1^*Cre*^/+ mice (*n* = 10 mice) exhibited severe to profound hearing loss. For comparison, the ABR thresholds of *Nlrp3*^+/+^; *Gfi1*^*Cre*/+^ mice (*n* = 4), *Nlrp3* ^*D*301*NneoR*^^/+^; *Gfi1*^+/+^ mice (*n* = 6) and *Nlrp3*^+/+^; *Gfi*^+/+^ mice (*n* = 4) were recorded at P20.

**Figure 1:**
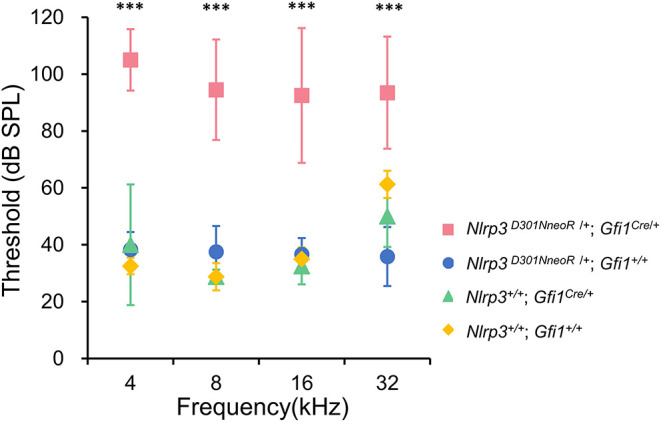
ABR thresholds in *Nlrp3* ^*D*301*NneoR*^^/+^*; Gfi1*^*Cre*/+^ mice (pink, *n* = 10 mice) were elevated compared to *Nlrp3* ^*D*301*NneoR*^^/+^*; Gfi1*^+/+^ mice (blue, *n* = 6 mice), *Nlrp3* ^+/+^*; Gfi1*^*Cre*/+^ mice (green, *n* = 4 mice), and *Nlrp3* ^+/+^*; Gfi1*^+/+^ mice (yellow, *n* = 4 mice) at P20. At each of the four tested frequencies, the threshold increases for *Nlrp3* ^*D*301*NneoR*^^/+^*; Gfi1*^*Cre*/+^ vs. *Nlrp3* ^*D*301*NneoR*^^/+^*; Gfi1*^+/+^, *Nlrp3* ^*D*301*NneoR*^^/+^*; Gfi1*^*Cre*/+^ vs. *Nlrp3*^+/+^*; Gfi1*^*Cre*/+^ and *Nlrp3* ^*D*301*NneoR*^^/+^*; Gfi1*^*Cre*/+^ vs. *Nlrp3*^+/+^*; Gfi1*^+/+^, were significant (Kruskal–Wallis test with the Dunn multiple-comparison test between all four groups). ABR, auditory brainstem response; P20, postnatal day 20. *** denotes *P* ≤ 0.001.

When compared to the ABR thresholds for *Nlrp3*^+/+^; *Gfi1*^*Cre*/+^ mice (four frequency average 37.8 ± 13.9 dB), *Nlrp3* ^*D*301*NneoR*^^/+^; *Gfi1*^+/+^ mice (37.1 ± 7.5) and *Nlrp3*^+/+^; *Gfi*^+/+^ mice (39.4 ± 13.8 dB), the ABR thresholds of the *Nlrp3* ^*D*301*NneoR*^^/+^; *Gfi*1^*Cre*^/+ mice (96.4 ± 20.4 dB) were significantly higher across the four tested frequencies (Figure 1, *P*≤0.05, Kruskal–Wallis test with the Dunn *post-hoc* test). The difference between the results of the *Nlrp3* ^*D*301*NneoR*^^/+^; *Gfi*1^*Cre*^/+ mice and the *Nlrp3*^+/+^; *Gfi1*^*Cre*/+^ mice means that the hearing loss observed in *Nlrp3* ^*D*301*NneoR*^^/+^; *Gfi*1^*Cre*^/+ mice was entirely due to the p.D301N variant and not due to *Gfi1* haploinsufficiency.

### Contrast-Enhanced MR Images Show Cochlear Inflammation of the *Nlrp3 ^*D*301*NneoR*^* ^/+^; *Gfi*1^*Cre*^/+ Mouse

**Figure 2:**
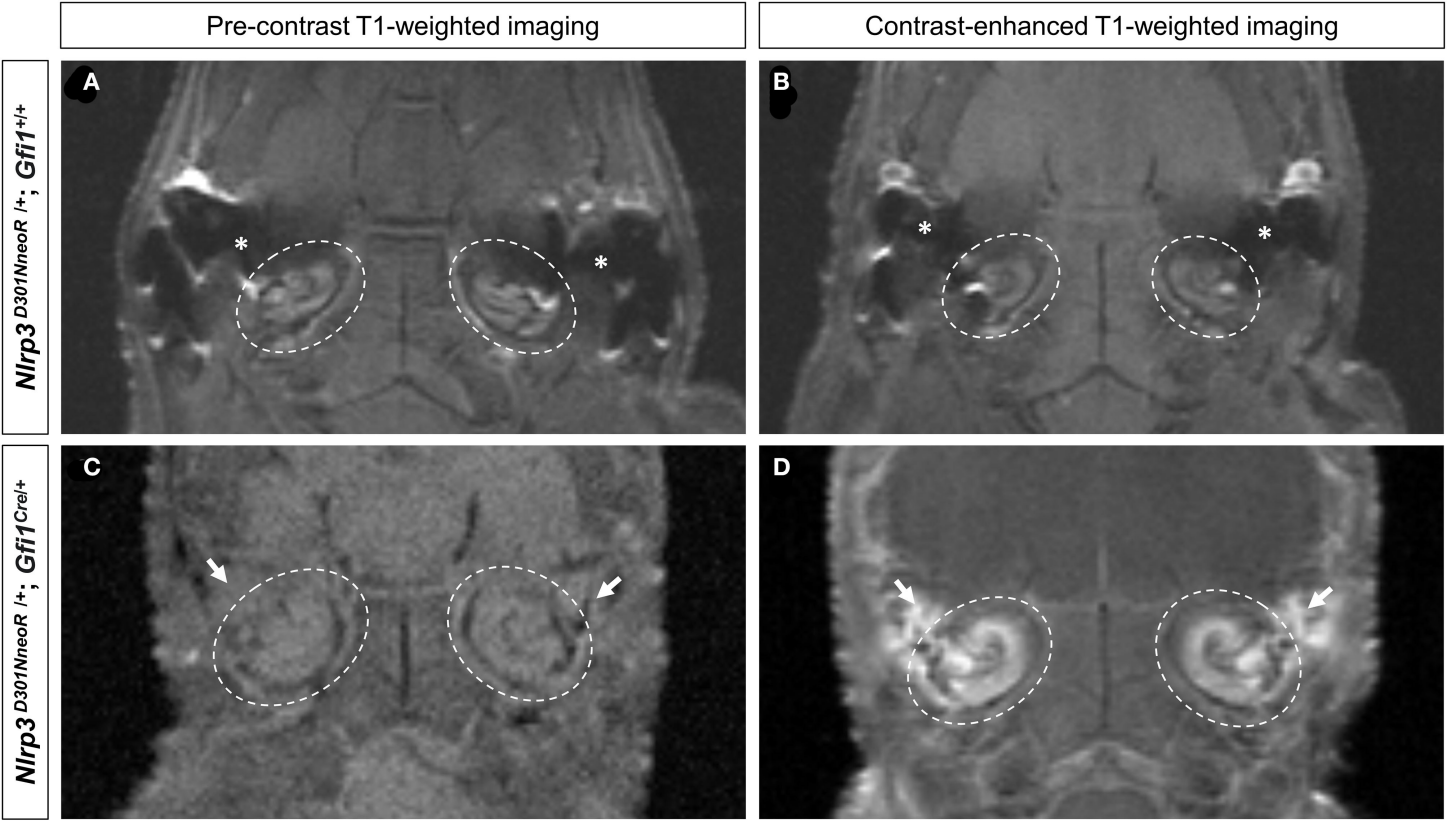
Pre- and post-contrast-enhanced T1-weighted images of the mouse head. Location of the cochlea is shown in dashed circles. The pre-contrast images of both the *Nlrp3* ^*D*301*NneoR*^^/+^; *Gfi*1^+^/+ **(A)** and the *Nlrp3* ^*D*301*NneoR*^^/+^; *Gfi*1^*Cre*^/+ mouse **(C)** show similar signal intensity of the cochlea to its surrounding tissue. Images taken 30 min after gadolinium injection, show enhancement of the cochlea in the *Nlrp3* ^*D*301*NneoR*^^/+^; *Gfi*1^*Cre*^/+ mouse **(D)** but not in the cochlea of the *Nlrp3* ^*D*301*NneoR*^^/+^; *Gfi*1^+^/+ mouse **(B)**. The *Nlrp3* ^*D*301*NneoR*^^/+^; *Gfi*1^*Cre*^/+ mouse also has inflammation in the middle ear (white arrows) where in the healthy condition should contain air and appear black as can be seen in the middle ear of the *Nlrp3* ^*D*301*NneoR*^^/+^; *Gfi*1^+^/+ mouse (asterisks).

The pre-contrast T1-weighted images of both the *Nlrp3*^*D*301*NneoR*/+^*; Gfi1*^+/+^ and the *Nlrp3*^*D*301*NneoR*/+^*; Gfi1*^*Cre*/+^ mouse showed similar signal intensity of the cochlea (Figures 2A,C). The same images were acquired after injection of contrast material into the tail vein which showed enhancement of the fluid compartment of the cochlea of the *Nlrp3*^*D*301*NneoR*/+^*; Gfi1*^*Cre*/+^ (Figure 2D). As for the *Nlrp3*^*D*301*NneoR*/+^*; Gfi1*^+/+^ mouse, there was no enhancement of the cochlea (Figure 2B).

Of note, middle ear inflammation was also visible on the pre- and post-contrast enhanced images of the *Nlrp3*^*D*301*NneoR*/+^*; Gfi1*^*Cre*/+^mouse (Figures 2C,D).

### Localization of Nlrp3 in the Inner Ear From *Nlrp3 ^*D*301*NneoR*^* ^/+^; *Gfi*1^*Cre*^/+ and *Nlrp3 ^*D*301*NneoR*^* ^/+^; *Gfi1^+/+^* Mice

**Figure 3:**
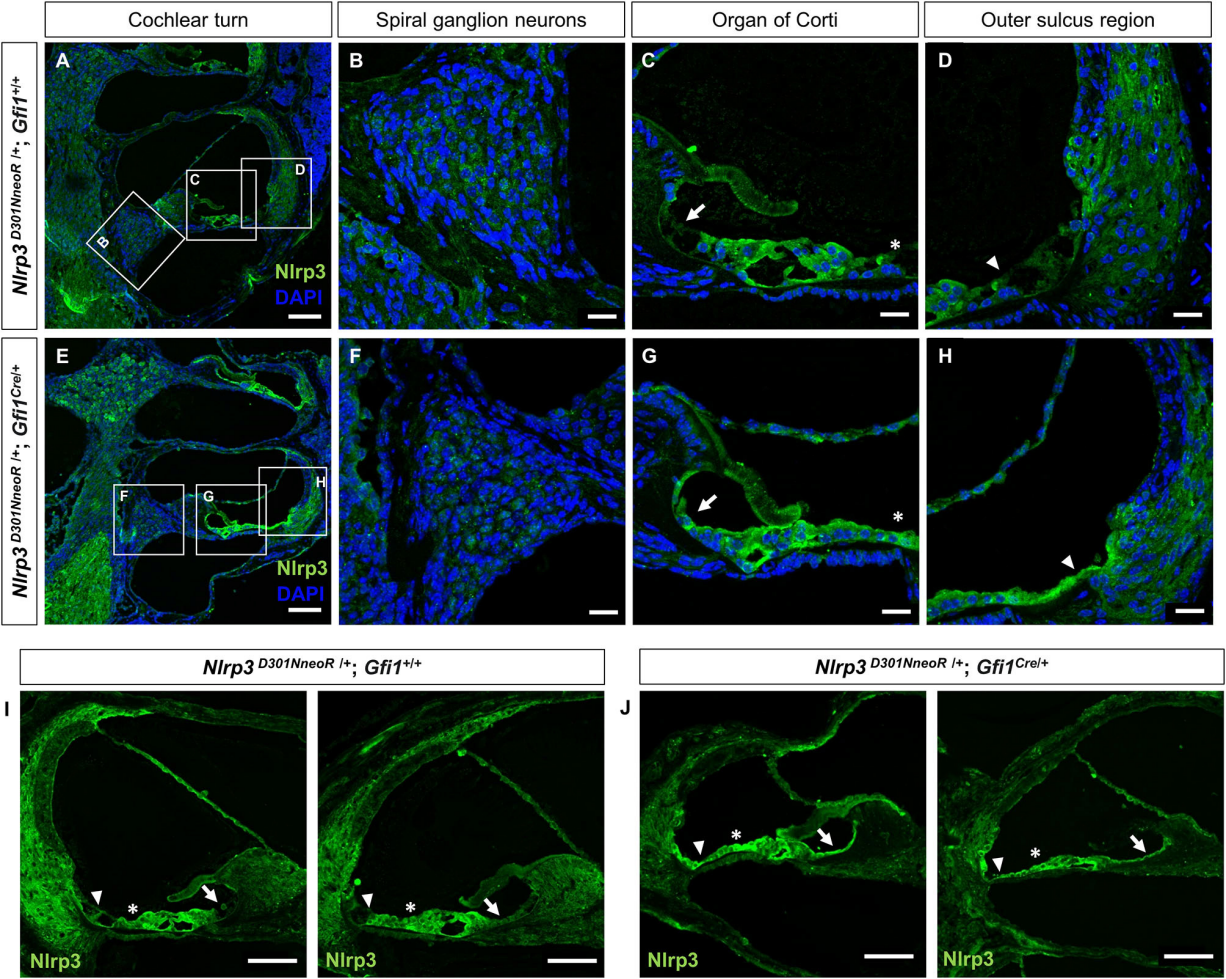
Inner ear section immunohistochemistry from P15 *Nlrp3* ^*D*301*NneoR*^^/+^; *Gfi1*^*Cre*/+^ and *Nlrp3* ^*D*301*NneoR*^^/+^; *Gfi*1^+^/+ mice stained with an anti-NLRP3 antibody. Nlrp3 was more widely overexpressed in the *Nlrp3* ^*D*301*NneoR*^^/+^; *Gfi*1^*Cre*^/+ mouse **(E–H)** than in the *Nlrp3* ^*D*301*NneoR*^^/+^*; Gfi1*^+/+^ mouse **(A–D)**. In both types of mice, Nlrp3 was expressed in the spiral prominence **(D,H)**, the organ of Corti **(C,G)**, and the spiral ganglion neurons **(B,F)**. A difference in the expression of Nlrp3 was noted in the inner sulcus cells (arrows), Hensen's cells (asterisks), and the outer sulcus region (arrowheads). Similar expression pattern of Nlrp3 was observed in two other cochlear turns of *Nlrp3* ^*D*301*NneoR*^^/+^; *Gfi1*^+/+^ **(I)** and *Nlrp3* ^*D*301*NneoR*^^/+^; *Gfi*1^*Cre*^/+ mice **(J)**. (*n* = 6 cochleae for *Nlrp3* ^*D*301*NneoR*^^/+^; *Gfi1*^*Cre*/+^ and *n* = 6 cochleae for *Nlrp3* ^*D*301*NneoR*^^/+^; *Gfi1*^+/+^) Nlrp3 in green, DAPI in blue, the scale bars represent 100 μm for **(A,E,I,J)** and 20 μm for **(B–D,F–H)**. P15, postnatal day 15.

**Figure 4:**
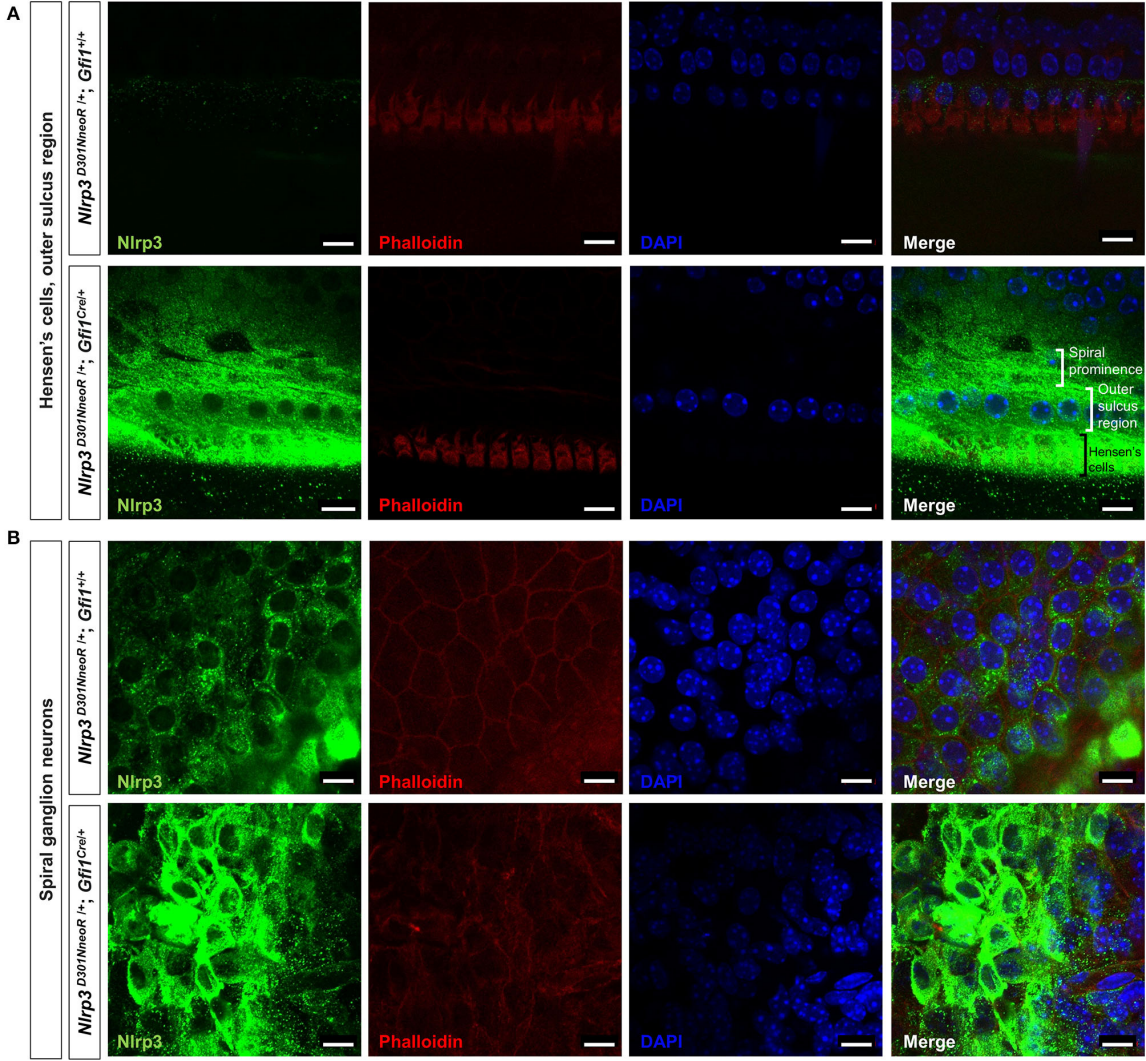
Whole-mount immunofluorescence of a P15 *Nlrp3* ^*D*301*NneoR*^^/+/+^; *Gfi1*^*Cre*/+^ mouse cochlea and a *Nlrp3* ^*D*301*NneoR*^^/+^; *Gfi*1^+^/+ mouse cochlea. Nlrp3 was overexpressed in the *Nlrp3* ^*D*301*NneoR*^^/+^; *Gfi*1^*Cre*^/+ mouse [bottom panels of **(A,B)**] compared to the *Nlrp3* ^*D*301*NneoR*^^/+^; *Gfi1*^+/+^ mouse [top panels of **(A,B)**] in Hensen's cells and the outer sulcus region **(A)** and the spiral ganglion neurons **(B)**. (*n* = 6 cochleae for *Nlrp3* ^*D*301*NneoR*^^/+^; *Gfi1*^*Cre*/+^ and *n* = 6 cochleae for *Nlrp3* ^*D*301*NneoR*^^/+^; *Gfi1*^+/+^) Nlrp3 in green, phalloidin in red, DAPI in blue, the scale bars represent 10 μm. P15, postnatal day 15.

To specify Nlrp3 expression in the cochlear tissue, we performed immunohistochemical staining using anti-NLRP3 antibody. The expression of Nlrp3 protein was evaluated in the cochleae of *Nlrp3* ^*D*301*NneoR*^^/+^*; Gfi1*^+/+^ and *Nlrp3* ^*D*301*NneoR*^^/+^; *Gfi*1^*Cre*^/+ mice at P15 (Figures 3, 4). In the cochlea of *Nlrp3* ^*D*301*NneoR*^^/+^*; Gfi1*^+/+^ mouse, which was expected to show wild-type expression of Nlrp3, Nlrp3 immunoreactivity was mainly concentrated in the spiral prominence (Figures 3A,D), the organ of Corti (Figures 3A,C), and the spiral ganglion neurons (Figures 3A,B). In the cochlea of *Nlrp3* ^*D*301*NneoR*^^/+^; *Gfi*1^*Cre*^/+ mouse, there appeared an overall overexpression of mutant Nlrp3, and the immunostaining was noted in the spiral prominence, the outer sulcus region (Claudius cells and outer sulcus cells) (Figures 3E,H), the organ of Corti, the inner sulcus (Figures 3E,G), and the spiral ganglion neurons (Figures 3E,H). Similar immunostaining patterns of Nlrp3 were seen throughout the whole cochlea along the tonotopic axis (Figures 3I,J). Noticeably, the Nlrp3 expression pattern was shown to differ the most in the outer sulcus region and the inner sulcus between the two types of mice.

Meanwhile, immunohistochemical staining of the whole mount preparations revealed similar but rather restricted expression pattern compared with those observed in the frozen section. The overexpression of mutant Nlrp3 compared to the pattern of wild-type Nlrp3 expression was most prominent at the Hensen's cells and the outer sulcus region (Figure 4A), as well as the spiral ganglion neurons (Figure 4B).

### Hair Cell Count

The number of inner and outer hair cells were quantified and compared in *Nlrp3* ^*D*301*NneoR*^^/+^*; Gfi1*^+/+^ mice (*n* = 6 cochleae) and *Nlrp3* ^*D*301*NneoR*^^/+^; *Gfi*1^*Cre*^/+ mice (*n* = 6 cochleae) at P15. Neither IHC nor OHC number showed significant difference in these two groups (Supplementary Figure 2). All inner and outer hair cells looked morphologically intact in *Nlrp3* ^*D*301*NneoR*^^/+^*; Gfi1*^*Cre*/+^ mice while the ABR results showed profound hearing loss in this group.

### Cochlear Histopathology in *Nlrp3 ^*D*301*NneoR*^* ^/+^; *Gfi*1^*Cre*^/+ Mice

**Figure 5:**
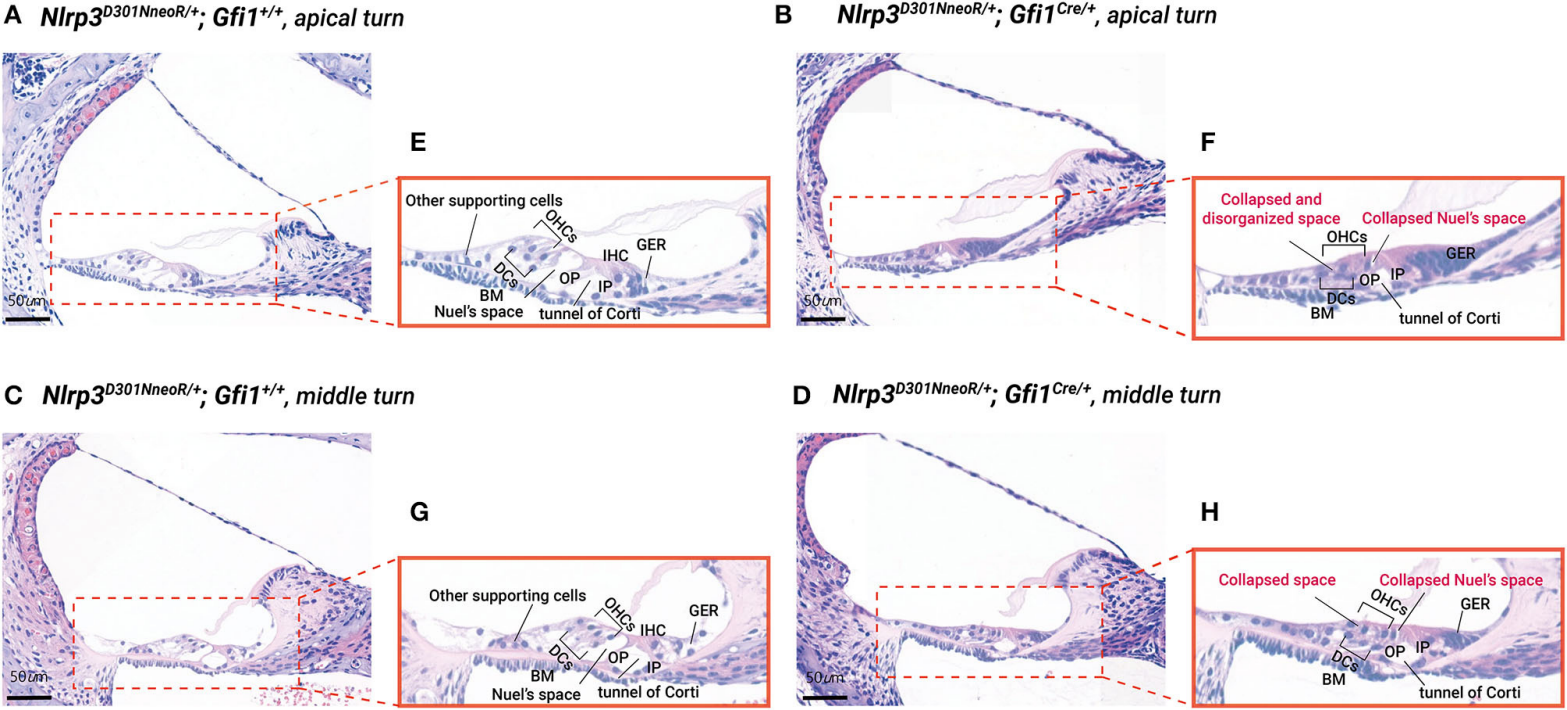
Cochlear morphology of *Nlrp3* ^*D*301*NneoR*^^/+^; *Gfi*1^*Cre*^/+ and *Nlrp3* ^*D*301*NneoR*^^/+^; *Gfi1*^+/+^ (normal phenotype) mice at P12. Representative hematoxylin and eosin-stained paraffin sections of *Nlrp3* ^*D*301*NneoR*^^/+^; *Gfi1*^+/+^ mouse [**(A)**, apex/**(C)**, middle] and *Nlrp3* ^*D*301*NneoR*^^/+^; *Gfi*1^*Cre*^/+ mice [**(B)**, apex/**(D)**, middle]. Close-ups of the organ of Corti (dotted box) from *Nlrp3* ^*D*301*NneoR*^^/+^; *Gfi1*^+/+^ mice **(E,G)** and *Nlrp3* ^*D*301*NneoR*^^/+^; *Gfi*1^*Cre*^/+ mice **(F,H)** are illustrated. Notice the apparent collapse of organ of Corti in *Nlrp3*^*D*301*N*/+^ mice (P12). P12, postnatal day 12; OHCs, outer hair cells; IHCs, inner hair cells; DCs, Dieter's cells; BM, basilar membrane; OP, outer pillar cells; IP, inner pillar cells; GER, greater epithelial ridge. The scale bars represent 500 mm.

**Figure 6:**
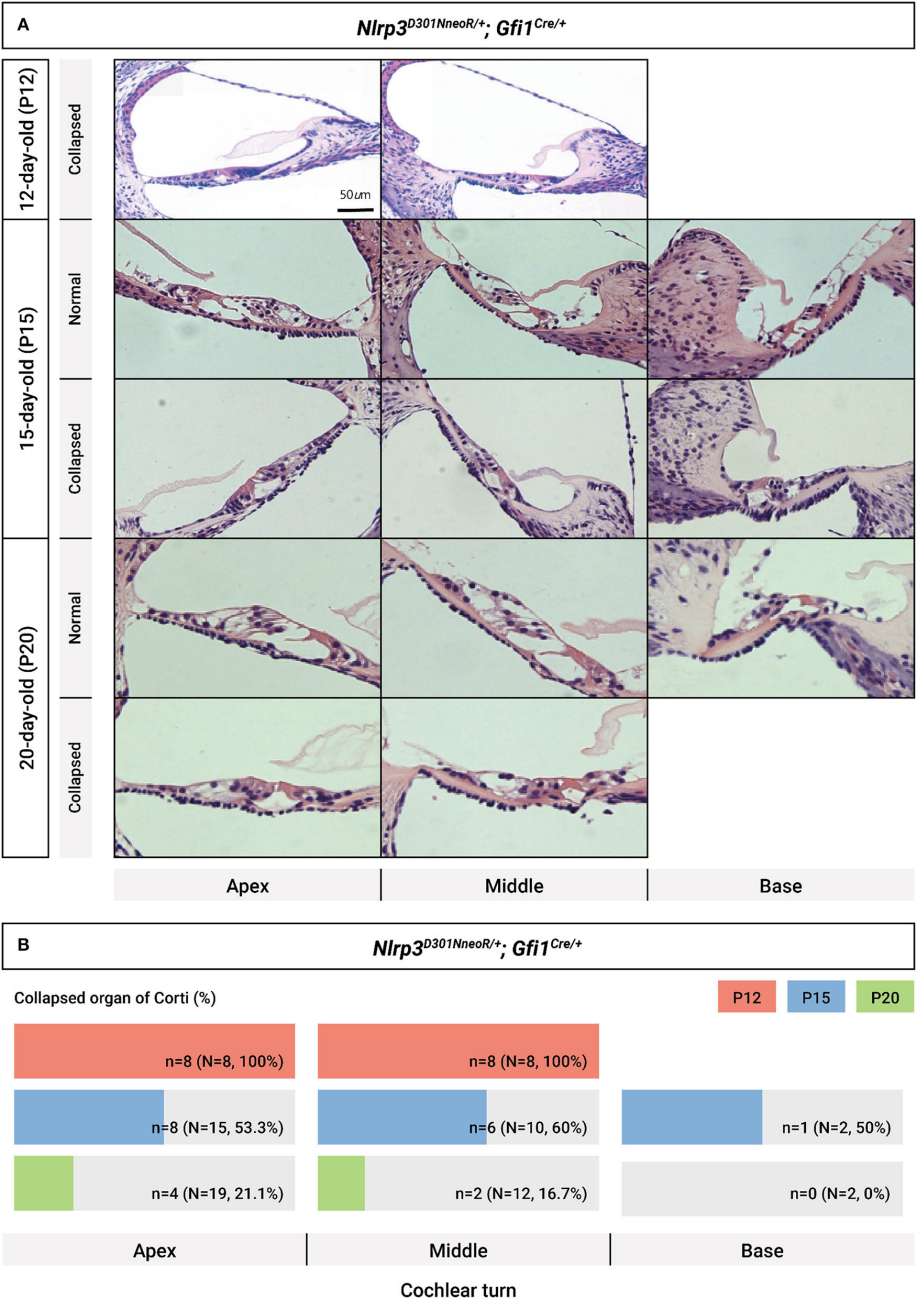
Chronological evaluation of cochlear morphology in *Nlrp3* ^*D*301*NneoR*^^/+^; *Gfi*1^*Cre*^/+ mice. **(A)** Representative sections from the cochlear turns obtained at P12, P15, and P20, respectively, are depicted (normal cochlear morphology vs. collapsed morphology of organ of Corti). **(B)** Proportion of collapsed organs of Corti at each time point, depending on cochlear turns. The scale bars represent 500 mm (P12) and 1,000 mm (P15 and P20). P, postnatal day.

A close evaluation of the organ of Corti demonstrated a difference in histopathology between *Nlrp3* ^*D*301*NneoR*^^/+^; *Gfi*1^*Cre*^/+ and *Nlrp3* ^*D*301*NneoR*^^/+^*; Gfi1*^+/+^ (normal phenotype) mice at P12 (Figure 5). At the middle and apical turns of the cochlea, hair and supporting cells were present in mice of both genotypes. However, *Nlrp3* ^*D*301*NneoR*^^/+^; *Gfi*1^*Cre*^/+ mice consistently exhibited a severely collapsed or disorganized organ of Corti between outer hair cells/supporting dieter's cell and the basilar membrane compared with *Nlrp3* ^*D*301*NneoR*^^/+^*; Gfi1*^+/+^mice, leading to an apparently collapsed Nuel's space (space between outer pillar cells and outer hair cells/dieter's cell). Additionally, nuclei of the greater epithelial ridge and other supporting cells close to the spiral ligament in *Nlrp3* ^*D*301*NneoR*^^/+^; *Gfi*1^*Cre*^/+ mice tended to be more condensed than in *Nlrp3* ^*D*301*NneoR*^^/+^*; Gfi1*^+/+^ mice. Meanwhile, no meaningful differences were found in histopathology between *Nlrp3*^+/+^*; Gfi1*^*Cre*/+^ and *Nlrp3* ^*D*301*NneoR*^^/+^*; Gfi1*^+/+^ mice, implying that the collapsed morphology of the organ of Corti was also not attributable to *Gfi1* haploinsufficiency (Supplementary Figure 3). Interestingly, the sections of *Nlrp3* ^*D*301*NneoR*^^/+^; *Gfi*1^*Cre*^/+ mice tended to gradually restore normal morphology of the organ of Corti at P15, as compared to P12, ultimately revealing normal cochlear morphology in most *Nlrp3*^*D*301*N*/+^; *Gfi*1^*Cre*^/+ mice at P20 (Figure 6A). The proportion of collapsed organ of Corti at P12 was 100%, which gradually decreased to 21.1% at the apex, 16.7% at the middle and 0% at the base at P20 (Figure 6B). Together, the data indicate that *Nlrp3* gain-of-function might predispose mice to a developmental delay of the organ of Corti, especially at the early postnatal stage.

### Systemic Inflammation in the Other Organs of *Nlrp3 ^*D*301*NneoR*^^/+^; Gfi1^*Cre*/+^* Mice

**Figure 7:**
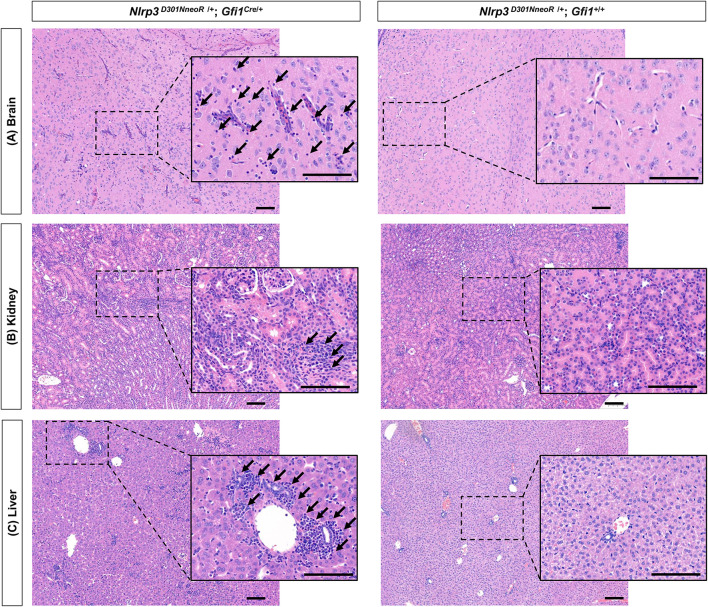
Generalized inflammation observed in several organs of *Nlrp*3^*D*301*NneoR*^/+; *Gfi*1^*Cre*^/+ mice compared to *Nlrp3* ^*D*301*NneoR*^^/+^; *Gfi1*^+/+^ mice. Representative image of inflammation at P20 in **(A)** brain (thalamus), **(B)** kidney, and **(C)** liver sections of *Nlrp3* ^*D*301*NneoR*^^/+^; *Gfi*1^*Cre*^/+ mice (left panels) compared to normal findings in *Nlrp3* ^*D*301*NneoR*/+^; *Gfi*1^+^/+ mice (right panels). Diffuse lymphocytic infiltration can be seen throughout the whole section. Insets show enlarged views of the dotted areas. Black arrows denote examples of areas with lymphocytic infiltration. P20, postnatal day 20. The scale bars represent 100 μm.

Various degrees of inflammation were observed on H&E-stained tissue slides of the brain, kidney, and liver (Figure 7). In detail, mild to moderate perivascular lymphocytic infiltration was observed in the brain parenchyma (representative image of the thalamus is shown in Figure 7), and increased inflammatory infiltrates were observed in the renal parenchyma. There was also moderate lymphocytic infiltration in the portal and lobular areas of the hepatic parenchyma. In the spleen, the lymphoid structure seems to be effaced, but definite inflammation was not seen. Definite inflammation was not observed in sections of the eye, stomach, arm, and leg.

## Discussion

We, for the first time, report the detailed quantifiable auditory and cochlear histopathologic phenotype of *Nlrp3* ^*D*301*NneoR*^^/+^; *Gfi*1^*Cre*^/+ mice. In this animal model, Nlrp3 expression was driven by the *Gfi1* promoter, which might be different from the endogenous Nlrp3 expression. Nonetheless, the *Nlrp3* ^*D*301*NneoR*^^/+^; *Gfi*1^*Cre*^/+ mouse is the first mouse model to show overexpression of Nlrp3 in the cochlea and potentially associated hearing loss, which could also provide a valuable tool to investigate the link between mutant Nlrp3 overexpression and hearing loss, further revealing the underlying mechanism of immune-related pathophysiology of autoinflammatory hearing loss.

Originally, we intended to generate inner ear-specific expression of *Nlrp3* (D301N) by crossing *Nlrp3* ^*D*301*NneoR*^^/+^ onto *Gfi1-Cre* knock-in mice, which was reported as a useful tool for inducing inner ear hair cell-specific expression of a target gene (15). However, *Gfi1* encodes a zinc-finger transcription factor that is also important for the development and maintenance of hematopoiesis, which could interfere with maturation of the immune system in the neonatal period and early infancy in mice. Accordingly, *Nlrp3* ^*D*301*NneoR*^^/+^; *Gfi*1^*Cre*^/+ mice in our study grew poorly (Supplementary Figure 4) and did not survive longer than 3 weeks (mean survival: 17.5 ± 2.7 days, *n* = 24), which might have been partly due to the intrinsic effect of systemic *Nlrp3* alterations corresponding to the phenotypes observed in human *NLRP3*-related autoinflammatory disorders, such as CINCA, which presents with fever, rash, joint symptoms, and central nervous system symptoms (19). However, its effects might be potentiated by systemic effects on several organs due to the abnormal development of the immune system, as was observed in comparisons of HE staining of several organs between *Nlrp3* ^*D*301*NneoR*^^/+^; *Gfi*1^*Cre*^/+ and *Nlrp3* ^*D*301*NneoR*^^/+^; *Gfi*1^+^/+ mice.

The widespread inflammation and very short life expectancy observed in the animal model may limit the impact of this study, which only yielded insights into the early changes of *Nlrp3* ^*D*301*NneoR*^^/+^; *Gfi*1^*Cre*^/+ mice in terms of systemic effects and developmental dysregulation of the cochlea and immune system. For instance, the number and morphology of hair cells of *Nlrp3* ^*D*301*NneoR*^^/+^; *Gfi*1^*Cre*^/+ mice were well-preserved not differing from the hair cells of the *Nlrp3* ^*D*301*NneoR*^^/+^; *Gfi*1^+^/+ mice. It is possible that the mice in this study did not survive long enough for hair cell damage from prolonged cochlear inflammation to manifest. Future studies using a different Cre mouse that generates milder phenotype and longer survival could show different outcomes. Based on the finding that only around 30% of patients with autoinflammatory inner ear disease respond to anakinra therapy (20) and the hearing in others deteriorate to the level that requires cochlear implantation, the damage caused by the inflammation on the cochlea can be progressive, irreversible and could lead to eventual hair cell damage.

The hearing thresholds of *Nlrp3* ^*D*301*NneoR*^^/+^; *Gfi*1^*Cre*^/+ mice were clearly worse than those of normal controls (Figure 1). A previous study regarding *Gfi*1^*Cre*^/+ mice demonstrated early-onset mild progressive hearing loss, which might serve as a source of bias when interpreting the ABR results (14). However, the difference in hearing thresholds between *Gfi*1^*Cre*^/+ mice and wild-type (*Gfi1*^+/+^) mice in that study was limited to 32 kHz until 2 months of life, meaning that intrinsic hearing deficit due to haploinsufficiency of *Gfi1* did not have a significant impact within <1 month of life. Accordingly, there was no difference in hearing thresholds between *Nlrp*3^+^/+; *Gfi*1^*Cre*^/+ mice and *Nlrp3* ^*D*301*NneoR*^^/+^; *Gfi1*^+/+^ mice (*P* > 0.05 at all frequencies by the Mann-Whitney test), although the mean hearing threshold of *Nlrp*3^+^/+; *Gfi*1^*Cre*^/+ mice at 32 kHz appeared slightly higher than those of *Nlrp3* ^*D*301*NneoR*^^/+^; *Gfi1*^+/+^ mice. On the contrary, *Nlrp3* ^*D*301*NneoR*^^/+^; *Gfi*1^*Cre*^/+ mice showed significantly worse hearing than mice expressing normal Nlrp3, irrespective of the loss of one copy of the *Gfi1*gene (Figure 1).

Middle ear inflammation observed in the MR images of the *Nlrp3* ^*D*301*NneoR*^^/+^; *Gfi*1^*Cre*^/+ mice could also have contributed to the elevated hearing thresholds. Indeed, prior studies of ABR threshold measurement in mouse models of otitis media have quantified at most 15 to 30 dB threshold shift depending on the tested frequency (21, 22). However, six out of the 10 *Nlrp3* ^*D*301*NneoR*^^/+^; *Gfi*1^*Cre*^/+ mice in this study did not show any ABR responses in all four tested frequencies even to the strongest stimuli (100 dB SPL). Thus, it is conceivable that a substantial portion of the elevation of hearing thresholds was due to the inner ear dysfunction, not the middle ear inflammation.

Cochlear inflammation was evident on the contrast-enhanced MR images of the *Nlrp3* ^*D*301*NneoR*^^/+^; *Gfi*1^*Cre*^/+ mouse. MRI studies of patients with CAPS or non-syndromic autoinflammatory inner ear disease have also identified cochlear enhancement (7–9). Intravenously injected contrast material can reach the inner ear via small vessels that supply the labyrinth and cochlea (23). Capillary networks present in the stria vascularis and spiral ligament form the blood-labyrinth barrier, composed of endothelial cells with tight-junctions surrounded by pericytes and resident macrophages (24). During inflammatory process of the cochlea, elevated levels of cytokines can increase capillary permeability thereby compromising the integrity of the blood-labyrinth barrier (25). The contrast material can enter the perilymphatic and endolymphatic spaces more freely than in the non-inflammatory state.

Immunohistochemical analyses using the anti-human NLRP3 polyclonal antibody revealed the widespread presence of Nlrp3, not limited to expression in the spiral ganglion, which has been reported in the literature (26). Rather, in the cochlea of *Nlrp3* ^*D*301*NneoR*^^/+^; *Gfi*1^*Cre*^/+ mice, Nlrp3 was expressed strongly in the spiral prominence, the organ of Corti, and the spiral ganglion neurons. *Nlrp*3^*D*301*NneoR*^/+; *Gfi*1^*Cre*^/+ mice carry one copy of a wild-type *Nlrp3* allele that would result in similar expression of Nlrp3 as seen in *Nlrp*3^*D*301*NneoR*^/+; *Gfi*1^+^/+ mice. The other floxed *Nlrp3* allele would lead to the overexpression of Nlrp3 in Cre-expressing cells. Although *Gfi1* is known to be expressed in hair cells in the late embryonic and postnatal inner ear (27), a previous validation of a *Gfi*1^*Cre*^/+ mouse model found that Cre-mediated recombination was not entirely hair cell-specific, but was also present in CD45+ monocytes/macrophages (14). A cell type-specific RNA-sequencing study of gene expression during mouse inner ear development also identified Gfi1 expression in the surrounding cells of the cochlea at P4 (28). Thus, in the current study, overexpression of Nlrp3 was observed in cells in which *Gfi1* was once expressed during the developmental period—that is, in hair cells and the regions of the cochlea where CD45+ cells can be found (in the organ of Corti, the basilar membrane, the spiral ligament, spiral limbus, and the neural region) (29).

The immunoreactivity to Nlrp3 in the cochlear sections differed the most in the inner sulcus and the outer sulcus region between the wildtype and mutant mice, displaying the significant staining exclusively from the mutant mice. The greatest difference in immunoreactivity to Nlrp3 in the cochlear sections between the wildtype and mutant mice was at the region of the inner sulcus and the outer sulcus, with significant positive staining occurring exclusively in the mutant mice. In the spiral prominence and the spiral ganglion neurons, both types of mice showed some degree of immunoreactivity, but it was stronger in the *Nlrp*3^*D*301*NneoR*^/+; *Gfi*1^*Cre*^/+ mice. The aforementioned areas in which we observed differences in immunoreactivity, we could attribute the findings to the ramifications of the gain-of-function variant of *Nlrp3*. Contrastingly, in the hair cells, the degree of Nlrp3 immunostaining was similar in both types of mice, precluding differentiation of the true immunostaining from staining artifacts. Absence of immunostaining of Nlrp3 from hair cells in the whole mount preparations—unlike in the frozen section—further complicated interpretation of immunostaining in the hair cells. This issue awaits further clarification through validation of the findings on a knockout mouse, which will be done in forthcoming studies.

Il-1β, a downstream product of Nlrp3 inflammasome activation (8), is known to form a positive feedback loop that potentially primes nearby cells in a paracrine manner (30, 31). In the inner sulcus and the outer sulcus region, Gfi1 expression was not anticipated, but overexpression of Nlrp3 was nonetheless evident. In these sites, the induced Il-1β from neighboring cells expressing mutant Nlrp3 could have enhanced the priming step to increase endogenous Nlrp3 expression. Future studies including co-localization of Nlrp3 with Il-1β and treatment with Il-1β antagonists would provide further insights.

Intriguingly, as was seen in Figure 6, the morphology of the organ of Corti in *Nlrp*3^*D*301*NneoR*^/+; *Gfi*1^*Cre*^/+ mice gradually normalized from P12 (significant difference between *Nlrp*3^*D*301*NneoR*^/+; *Gfi*1^*Cre*^/+ and *Nlrp*3^*D*301*NneoR*^/+; *Gfi*1^+^/+ mice) to P20, which might suggest that the effect of *Nlrp*3^*D*301*NneoR*^/+ on the development of inner ear is transient in the early postnatal period and further indicate that the elevation of the hearing threshold might not be solely due to the anatomical alterations. In further research, we need to generate a new mouse model by breeding Cre mice of specific immune cell population with *Nlrp3* knock-in mice, which could express Nlrp3, thereby coming one step closer to endogenous Nlrp3 expression. By doing so, we could elucidate the specific immune cell population associated with the development of autoinflammatory hearing loss in an animal model with a longer lifespan, which could enable a more detailed characterization of hearing loss and immune system dysregulation.

## Conclusion

*Nlrp3* ^*D*301*NneoR*^^/+^; *Gfi*1^*Cre*^/+ mice, an animal model of Nlrp3 expression driven by the *Gfi1* promoter, is the first mouse model to show quantifiable hearing loss and overexpression of Nlrp3 in the cochlea. This overexpression is potentially associated with hearing loss, and this model could also provide a valuable tool to investigate the link between mutant Nlrp3 overexpression and hearing loss, further revealing the underlying mechanism of inflammasome activation-mediated hearing loss.

## Data Availability Statement

The original contributions presented in the study are included in the article/Supplementary Material, further inquiries can be directed to the corresponding author/s.

## Ethics Statement

The animal study was reviewed and approved by Institutional Animal Care and Use Committee of Seoul National University Bundang Hospital.

## Author Contributions

BK and BC contributed to the conception and design of the study. YK, MK, and JH organized the data and conducted the experiments. YK, S-YL, JK, BK, and BC performed the data analysis. YK, S-YL, BK, and BC wrote the first draft of the manuscript. All authors contributed to manuscript revision, read, and approved the submitted version.

## Funding

This study was supported by the Basic Science Research Program through the NRF funded by the Ministry of Education (Grants 2021R1A2C2092038 and 2018R1A2B2001054 to BC), the Korea Government (MSIT) (No. 2021R1C1C1007980 to BK), also by SNUBH Intramural Research Funds (16-2020-0005 and 14-2021-0003 to BC), and the Institute of Information Communication Technology Planning and Evaluation (IITP) grant funded by the Korea Government (MSIT) [No. 2020-0-01441, Artificial Intelligence Convergence Research Center (Chungnam National University) to BK].

## Conflict of Interest

The authors declare that the research was conducted in the absence of any commercial or financial relationships that could be construed as a potential conflict of interest.

## Publisher's Note

All claims expressed in this article are solely those of the authors and do not necessarily represent those of their affiliated organizations, or those of the publisher, the editors and the reviewers. Any product that may be evaluated in this article, or claim that may be made by its manufacturer, is not guaranteed or endorsed by the publisher.

## Supplementary Material

Supplementary Figure 1 Validation of the anti-NLRP3 antibody on human embryonic kidney (HEK) 293 cells transfected with NLRP3 (Myc-DDK-tagged) cDNA. The anti-NLRP3 antibody showed specific binding to the HEK 293 cells transfected with NLRP3 (red color in the bottom panel) and not to the mock transfection control (top panel).

Supplementary Figure 2 Hair cell count between *Nlrp3* ^*D*301*NneoR*^^/+/+^; *Gfi1*^*Cre*/+^ mice and *Nlrp3* ^*D*301*NneoR*^^/+^; *Gfi*1^+^/+ mice. Number of inner **(A)** and outer **(B)** hair cells in segments spanning 1% of the whole cochlear length in P15 *Nlrp3* ^*D*301*NneoR*^^/+/+^; *Gfi1*^*Cre*/+^ mice (*n* = 6 cochleae) and *Nlrp3* ^*D*301*NneoR*^^/+^; *Gfi*1^+^/+ mice (*n* = 6 cochleae) were not different.

Supplementary Figure 3 Cochlear morphology of *Nlrp3*^+/+^; *Gfi1*^*Cre*/+^ mice at P15. Sections of the cochlea of *Nlrp3*^+/+^; *Gfi1*^*Cre*/+^ mice did not show collapsed morphology of the organ of Corti, unlike the *Nlrp3*^*D*301*N*/+^*; Gfi1*^*Cre*/+^ mice. P15, postnatal day 15.

Supplementary Figure 4 Differences in the morphology of *Nlrp3*^*D*301*N*/+^; *Gfi1*^+/+^ mice compared to *Nlrp3*^*D*301*N*/+^; *Gfi1*^*Cre*/+^ mice. At P11, two *Nlrp3* ^*D*301*NneoR*^^/+^; *Gfi*1^*Cre*^/+ mice were smaller (2.8 g and 3.1 g vs. 6.8 g) and showed more extensive areas of skin inflammation with hair loss than the *Nlrp3*^*D*301*N*/+^; *Gfi1*^+/+^mouse (left). P11, postnatal day 11.

## References

[1] Agostini L Martinon F Burns K McDermott MF Hawkins PN Tschopp JJI. Nalp3 forms an Il-1β-processing inflammasome with increased activity in Muckle-Wells autoinflammatory disorder. Immunity. (2004) 20:319–25. 10.1016/S1074-7613(04)00046-9 15030775

[2] Sutterwala FS Ogura Y Szczepanik M Lara-Tejero M Lichtenberger GS Grant EP. Critical role for Nalp3/Cias1/cryopyrin in innate and adaptive immunity through its regulation of caspase-1. Immunity. (2006) 24:317–27. 10.1016/j.immuni.2006.02.004 16546100

[3] Guarda G Zenger M Yazdi AS Schroder K Ferrero I Menu P. Differential expression of Nlrp3 among hematopoietic cells. J Immunol. (2011) 186:2529–34. 10.4049/jimmunol.1002720 21257968

[4] Martinon F Burns K Tschopp J. The inflammasome: a molecular platform triggering activation of inflammatory caspases and processing of ProIL-?. Mol Cell. (2002) 10:417–26. 10.1016/S1097-2765(02)00599-3 12191486

[5] Feldmann J Prieur AM Quartier P Berquin P Certain S Cortis E. Chronic infantile neurological cutaneous and articular syndrome is caused by mutations in Cias1, a gene highly expressed in polymorphonuclear cells and chondrocytes. Am J Hum Genet. (2002) 71:198–203. 10.1086/341357 12032915 PMC384980

[6] Kuemmerle-Deschner JB Koitschev A Ummenhofer K Hansmann S Plontke SK Koitschev C. Hearing loss in Muckle-Wells syndrome. Arthritis Rheum. (2013) 65:824–31. 10.1002/art.37810 23440695

[7] Ahmadi N Brewer CC Zalewski C King KA Butman JA Plass N. Cryopyrin-associated periodic syndromes: otolaryngologic and audiologic manifestations. Otolaryngol Head Neck Surg. (2011) 145:295–302. 10.1177/0194599811402296 21493283 PMC3407887

[8] Nakanishi H Kawashima Y Kurima K Chae JJ Ross AM Pinto-Patarroyo G. Nlrp3 mutation and cochlear autoinflammation cause syndromic and nonsyndromic hearing loss Dfna34 responsive to Anakinra therapy. Proc Natl Acad Sci USA. (2017) 114:E7766–75. 10.1073/pnas.1702946114 28847925 PMC5604003

[9] Kim BJ Kim YH Lee S Han JH Lee SY Seong J. Otological aspects of Nlrp3-related autoinflammatory disorder focusing on the responsiveness to Anakinra. Rheumatology. (2021) 60:1523–32. 10.1093/rheumatology/keaa511 33020839

[10] Kelley N Jeltema D Duan Y He YJ. The Nlrp3 inflammasome: an overview of mechanisms of activation and regulation. Int J Mol Sci. (2019) 20:3328. 10.3390/ijms20133328 31284572 PMC6651423

[11] Aksentijevich I Nowak M Mallah M Chae JJ Watford WT Hofmann SR. *De novo* Cias1 mutations, cytokine activation, and evidence for genetic heterogeneity in patients with neonatal-onset multisystem inflammatory disease (Nomid): a new member of the expanding family of Pyrin-associated autoinflammatory diseases. Arthritis Rheum. (2002) 46:3340–8. 10.1002/art.10688 12483741 PMC4556432

[12] Kim YH Kim BJ Han J Choi BY Lee S. Long-term efficacy of Anakinra in cryopyrin-associated periodic syndrome: focus on destructive arthropathy. J Clin Immunol. (2021) 41:1936–9. 10.1007/s10875-021-01099-z 34346012

[13] Kim BJ Kim YH Han JH Lee SY Carandang M Lee DH. Outcome of cochlear implantation in Nlrp3-related autoinflammatory inner ear disorders. Otol Neurotol. (2021) 42:e168–71. 10.1097/MAO.0000000000002933 33156237

[14] Matern M Vijayakumar S Margulies Z Milon B Song Y Elkon R. Gfi1cre mice have early onset progressive hearing loss and induce recombination in numerous inner ear non-hair cells. Sci Rep. (2017) 7:1–13. 10.1038/srep42079 28181545 PMC5299610

[15] Yang H Gan J Xie X Deng M Feng L Chen X. Gfi1-Cre knock-in mouse line: a tool for inner ear hair cell-specific gene deletion. Genesis. (2010) 48:400–6. 10.1002/dvg.20632 20533399 PMC2885521

[16] Cox BC Liu Z Lagarde MMM Zuo J. Conditional gene expression in the mouse inner ear using Cre-Loxp. J Assoc Res Otolaryngol. (2012) 13:295–322. 10.1007/s10162-012-0324-5 22526732 PMC3346893

[17] Akil O Oursler A Fan K Lustig LR. Mouse auditory brainstem response testing. Bio Protoc. (2016) 6:e1768. 10.21769/BioProtoc.1768 28280753 PMC5340198

[18] Viberg A Canlon B. The guide to plotting a cochleogram. Hear Res. (2004) 197:1–10. 10.1016/j.heares.2004.04.016 15504598

[19] Bonar SL Brydges SD Mueller JL McGeough MD Pena C Chen D. Constitutively activated Nlrp3 inflammasome causes inflammation and abnormal skeletal development in mice. PLoS ONE. (2012) 7:e35979. 10.1371/journal.pone.0035979 22558291 PMC3338787

[20] Sibley CH Plass N Snow J Wiggs EA Brewer CC King KA. Sustained response and prevention of damage progression in patients with neonatal-onset multisystem inflammatory disease treated with anakinra: a cohort study to determine three-and five-year outcomes. Arthritis Rheum. (2012) 64:2375–86. 10.1002/art.34409 22294344 PMC3474541

[21] Han F Yu H Zhang J Tian C Schmidt C Nava C. Otitis media in a mouse model for down syndrome. Int J Exp Pathol. (2009) 90:480–8. 10.1111/j.1365-2613.2009.00677.x 19765102 PMC2768146

[22] MacArthur CJ Hefeneider SH Kempton JB Parrish SK McCoy SL Trune DR. Evaluation of the mouse model for acute otitis media. Hear Res. (2006) 219:12–23. 10.1016/j.heares.2006.05.012 16887307

[23] Song CI Pogson JM Andresen NS Ward BK. Mri with gadolinium as a measure of blood-labyrinth barrier integrity in patients with inner ear symptoms: a scoping review. Front Neurol. (2021) 12:746. 10.3389/fneur.2021.662264 34093410 PMC8173087

[24] Zhang W Dai M Fridberger A Hassan A DeGagne J Neng L. Perivascular-resident macrophage-like melanocytes in the inner ear are essential for the integrity of the intrastrial fluid–blood barrier. Proc Natl Acad Sci USA. (2012) 109:10388–93. 10.1073/pnas.1205210109 22689949 PMC3387119

[25] Ichimiya I Yoshida K Hirano T Suzuki M Mogi G. Significance of spiral ligament fibrocytes with cochlear inflammation. Int J Pediatr Otorhinolaryngol. (2000) 56:45–51. 10.1016/S0165-5876(00)00408-0 11074115

[26] Chen P He L Pang X Wang X Yang T Wu H. Nlrp3 is expressed in the spiral ganglion neurons and associated with both syndromic and nonsyndromic sensorineural deafness. Neural Plast. (2016) 2016:3018132. 10.1155/2016/3018132 27965898 PMC5124661

[27] Wallis D Hamblen M Zhou Y Venken KJ Schumacher A Grimes HL. The zinc finger transcription factor Gfi1, implicated in lymphomagenesis, is required for inner ear hair cell differentiation and survival. Development. (2003) 130:221–32. 10.1242/dev.00190 12441305

[28] Scheffer DI Shen J Corey DP Chen Z-Y. Gene expression by mouse inner ear hair cells during development. J Neurosci. (2015) 35:6366–80. 10.1523/JNEUROSCI.5126-14.2015 25904789 PMC4405555

[29] Dong Y Zhang C Frye M Yang W Ding D Sharma A. Differential fates of tissue macrophages in the cochlea during postnatal development. Hear Res. (2018) 365:110–26. 10.1016/j.heares.2018.05.010 29804721 PMC6026078

[30] Gritsenko A Green JP Brough D Lopez-Castejon G. Mechanisms of Nlrp3 priming in inflammaging and age related diseases. Cytokine Growth Factor Rev. (2020) 55:15–25. 10.1016/j.cytogfr.2020.08.003 32883606 PMC7571497

[31] Hiscott J Marois J Garoufalis J D'addario M Roulston A Kwan I. Characterization of a functional Nf-Kappa B site in the human interleukin 1 beta promoter: evidence for a positive autoregulatory loop. Mol Cell Biol. (1993) 13:6231–40. 10.1128/mcb.13.10.6231-6240.1993 8413223 PMC364682

