# Supplementary figures and images for: Auditory Phenotype and Histopathologic Findings of a Mutant Nlrp3 Expression Mouse Model
Source: Front Neurol. 2022 Jun 24;13:890256. doi: 10.3389/fneur.2022.890256 (PMC9263128; doi:10.3389/fneur.2022.890256)

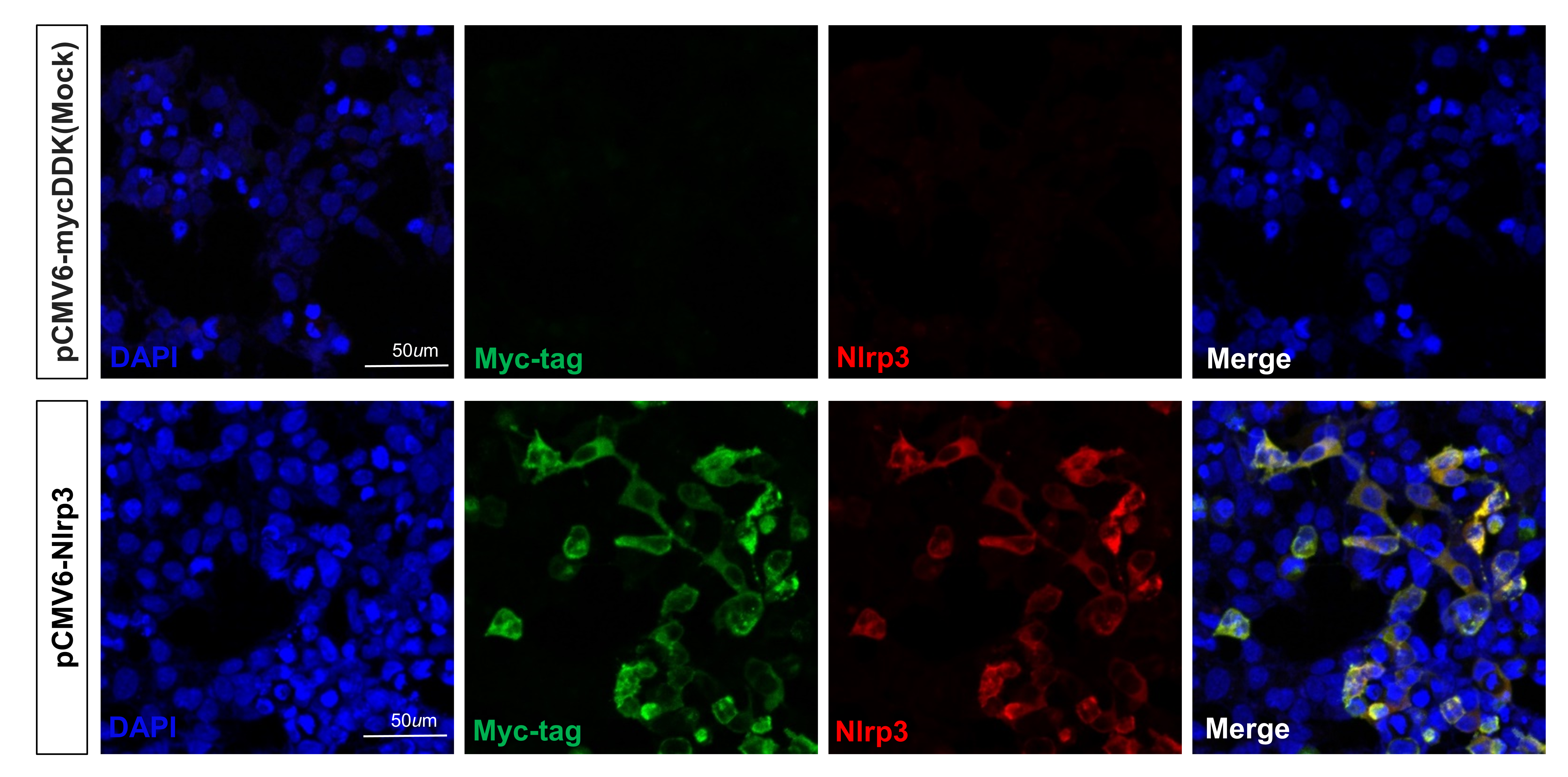

Supplement: Supplementary Figure 1 — Validation of the anti-NLRP3 antibody on human embryonic kidney (HEK) 293 cells transfected with NLRP3 (Myc-DDK-tagged) cDNA. The anti-NLRP3 antibody showed specific binding to the HEK 293 cells transfected with NLRP3 (red color in the bottom panel) and not to the mock transfection control (top panel). [file Image_1.TIF]

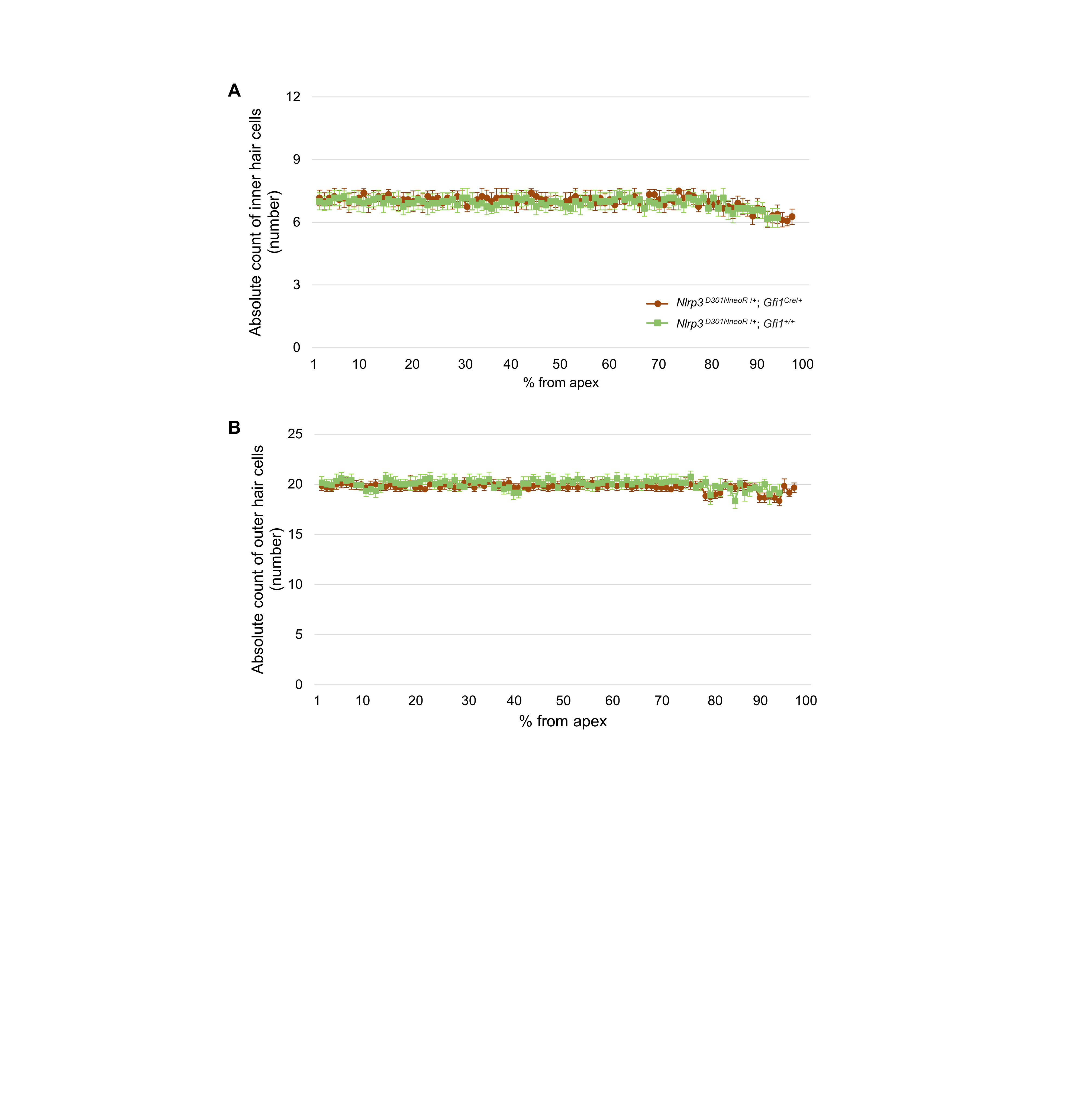

Supplement: Supplementary Figure 2 — Hair cell count between Nlrp3 D301NneoR /+/+; Gfi1Cre/+ mice and Nlrp3 D301NneoR /+; Gfi1+/+ mice. Number of inner (A) and outer (B) hair cells in segments spanning 1% of the whole cochlear length in P15 Nlrp3 D301NneoR /+/+; Gfi1Cre/+ mice (n = 6 cochleae) and Nlrp3 D301NneoR /+; Gfi1+/+ mice (n = 6 cochleae) were not different. [file Image_2.TIF]

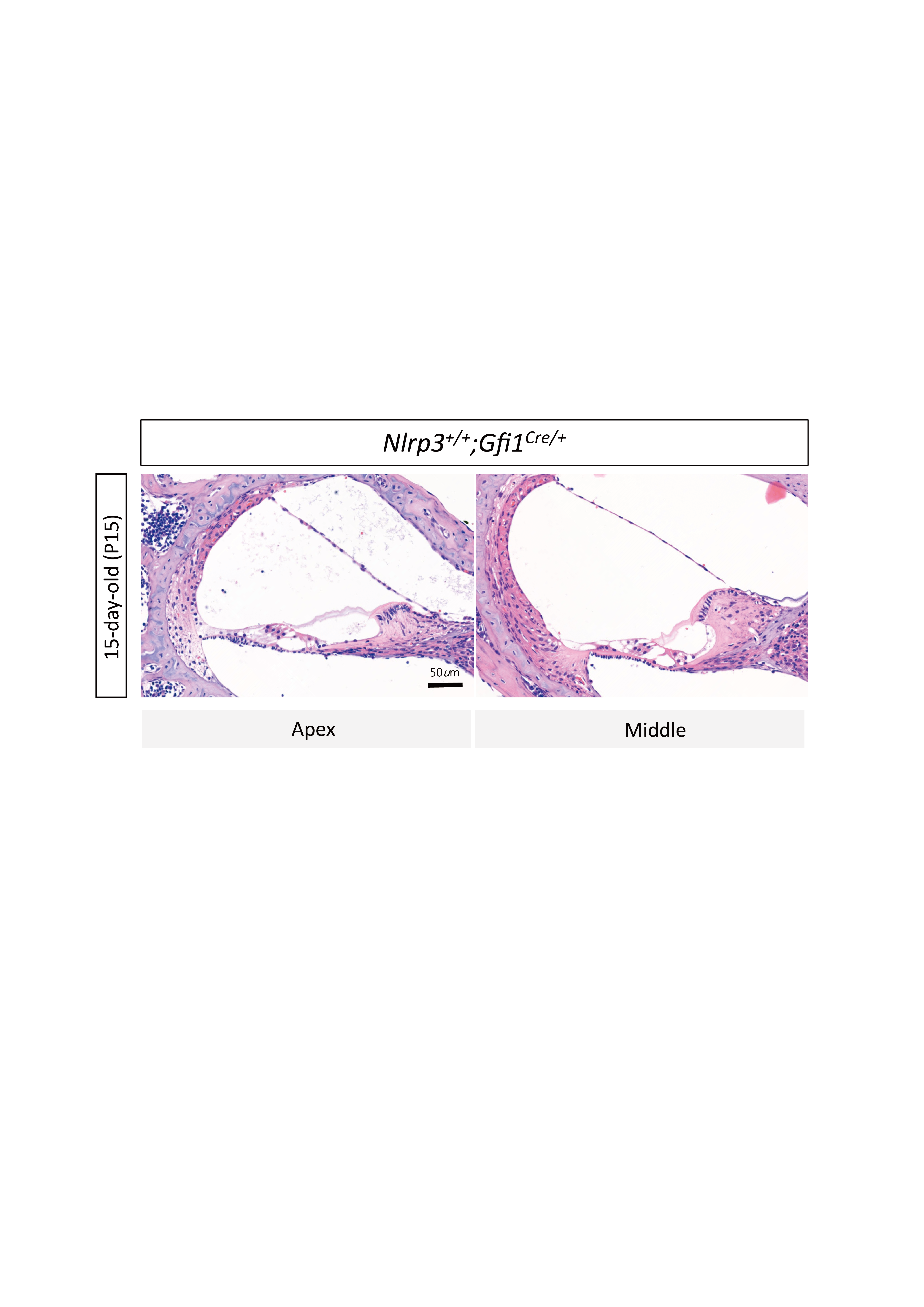

Supplement: Supplementary Figure 3 — Cochlear morphology of Nlrp3+/+; Gfi1Cre/+ mice at P15. Sections of the cochlea of Nlrp3+/+; Gfi1Cre/+ mice did not show collapsed morphology of the organ of Corti, unlike the Nlrp3D301N/+; Gfi1Cre/+ mice. P15, postnatal day 15. [file Image_3.PNG]

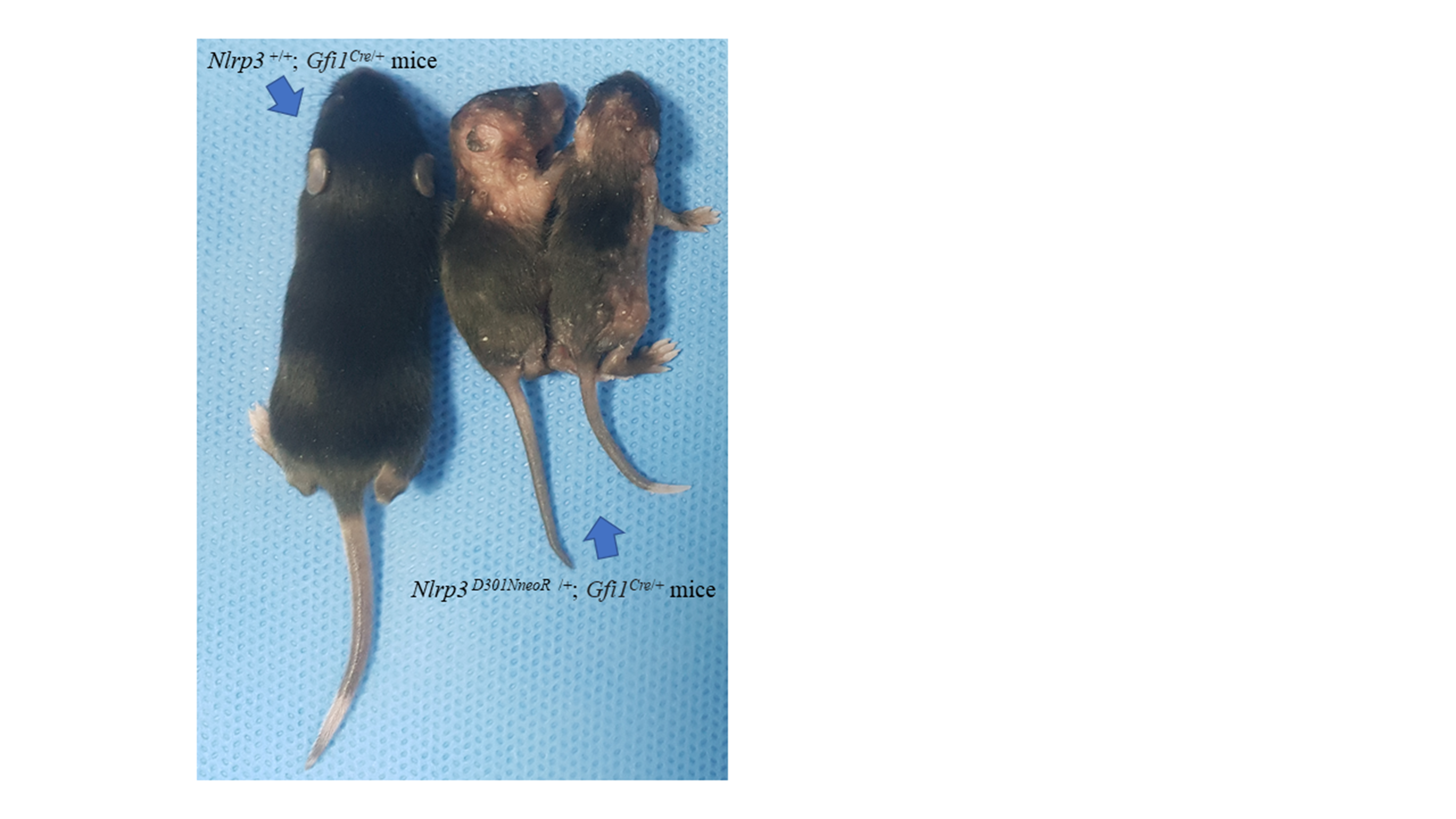

Supplement: Supplementary Figure 4 — Differences in the morphology of Nlrp3D301N/+; Gfi1+/+ mice compared to Nlrp3D301N/+; Gfi1Cre/+ mice. At P11, two Nlrp3 D301NneoR /+; Gfi1Cre/+ mice were smaller (2.8 g and 3.1 g vs. 6.8 g) and showed more extensive areas of skin inflammation with hair loss than the Nlrp3D301N/+; Gfi1+/+mouse (left). P11, postnatal day 11. [file Image_4.TIF]
